# The Operational Feasibility of Vaccination Programs Targeting Influenza Risk Groups in the World Health Organization (WHO) African and South-East Asian Regions

**DOI:** 10.1093/cid/ciab393

**Published:** 2021-05-04

**Authors:** Justin R Ortiz, Stephen L Yu, Amanda J Driscoll, Sarah R Williams, Joanie Robertson, Jui-Shan Hsu, Wilbur H Chen, Robin J Biellik, Samba Sow, Sonali Kochhar, Kathleen M Neuzil

**Affiliations:** 1 Center for Vaccine Development and Global Health, University of Maryland School of Medicine, Baltimore, Maryland, USA; 2 Division of Pulmonary and Critical Care Medicine, University of Maryland School of Medicine, Baltimore, Maryland, USA; 3 PATH, Seattle, Washington, USA; 4 Independent Consultant, La Rippe, Switzerland; 5 Centre pour le Développement des Vaccins, Ministère de la Santé, Bamako, Mali; 6 Global Healthcare Consulting, New Delhi, India; 6a and Department of Global Health, Seattle, Washington, USA

**Keywords:** influenza vaccine, Africa, South-East Asia, immunization, influenza

## Abstract

**Background:**

Influenza vaccination is uncommon in low-resource settings. We evaluated aspects of operational feasibility of influenza vaccination programs targeting risk groups in the World Health Organization (WHO) African (AFR) and South-East Asian (SEAR) Regions.

**Methods:**

We estimated routine immunization and influenza vaccination campaign doses, doses per vaccinator, and cold storage requirements for 1 simulated country in each region using evidence-based population distribution, vaccination schedule, and vaccine volumes. Influenza vaccination targeted persons <5 years, pregnant women, persons with chronic diseases, persons ≥65 years, and healthcare workers (HCW). For the AFR country, we compared vaccine volumes to actual storage capacities.

**Results:**

Targeting HCW had a small operational impact, and subsequent findings exclude this group. During 3-month influenza vaccination campaigns, monthly doses delivered in the AFR country increased from 15.0% for ≥65 years to 93.1% for <5 years and in the SEAR country from 19.6% for pregnant women to 145.0% for persons with chronic diseases. National-level cold storage capacity requirements increased in the AFR country from 4.1% for ≥65 years to 20.3% for <5 years and in the SEAR country from 3.9% for pregnant women to 28.8% for persons with chronic diseases. Subnational-level cold storage capacity requirements increased in the AFR country from 5.9% for ≥65 years to 36.8% for <5 years and the SEAR country from 17.6% for pregnant women to 56.0% for persons with chronic diseases.

**Conclusions:**

Influenza vaccination of most risk groups will require substantial increases in doses, doses per vaccinator, and cold storage capacity in countries where infrastructure and resources are limited.

## Teaser

We investigated the operational feasibility of preseasonal influenza vaccination campaigns in countries in World Health Organization (WHO) African and South-East Asian Regions. Influenza vaccination campaigns targeting most risk groups substantially increase doses and doses per vaccinator over routine immunization baselines.

The World Health Organization (WHO) recommends that persons at risk for severe influenza illness receive influenza vaccine annually [1]. Despite this policy recommendation, influenza vaccines are not commonly used in much of the world [2–4]. Among the 83 low-income countries (LICs) and lower-middle-income countries (LMICs) in 2014, only 20 (24.1%) reported having national influenza vaccination policies. The WHO Regions with the lowest proportions of countries with influenza vaccination policies in 2014 were the African Region (3/47) and the South-East Asian Region (2/11) [2].

There are many challenges to influenza vaccination programs that particularly affect LICs and LMICs, including the need for annual immunization, frequent formulation updates, short shelf-lives, targeted populations outside traditional vaccination contact ages, and unclear program impact on severe disease [5, 6]. Vaccine delivery and immunization supply chain limitations may also hinder influenza vaccination program implementation. In 2014, WHO noted that many country systems lacked sufficient capacity to accommodate the expansion of immunization programs [7]. Since then, a concerted global effort has improved supply chain infrastructure [8], but substantial vaccine storage capacity limitations remain [9]. Immunization system strengthening has been a focus of the global severe acute respiratory syndrome coronavirus 2 (SARS-CoV-2) pandemic response; however, as of late 2020, the United Nations expressed concern about the low levels of readiness among LICs and LMICs to deliver pandemic vaccines [10].

In this study, we evaluate the anticipated impact influenza vaccination programs would have on vaccine delivery, workload, and cold storage in countries in the WHO African and South-East Asian Regions. Our objective was to assess the operational feasibility of influenza vaccination programs targeting risk groups in these regions.

## METHODS

### Study Design

This study is based on cold chain capacity assessments performed routinely by countries in preparation for new vaccine introductions [11]. We simulated immunization operations in 2 different countries of 20 million people in the WHO African and South-East Asian Regions, subsequently referred to as “African country” and “Asian country.” We applied the 2017 population age distributions for each region to each country [12]. All simulation inputs reflected regional averages, unless otherwise specified. Our outcomes of interest were the monthly percentage increases in vaccine doses and vaccine cold storage volumes during preseasonal influenza vaccination campaigns targeting risk groups.

### Immunization Schedules

We developed routine and influenza vaccination schedules for the simulated countries based on WHO policy recommendations (Table 1) [1, 13]. The influenza vaccination schedules targeted risk groups defined by WHO: children <5 years, pregnant women, the elderly, persons with chronic medical conditions (chronic diseases), and healthcare workers (HCWs) (Supplementary Table 1) [1]. We estimated the number of pregnant women from the 2017 birth cohort adjusted by assuming 15% of all pregnancies end in stillbirth or miscarriage [14]. Although WHO recommends that persons with “specific medical conditions” receive annual influenza vaccination, these conditions are not elaborated [1]. Unable to find prevalence estimates of persons with chronic diseases at high risk for severe influenza illness, we instead used prevalence estimates of conditions which increase the risk for severe SARS-CoV-2 illness by age category and WHO Region [15]. These conditions overlap considerably with influenza risk factors as defined by the US Centers for Disease Control and Prevention [15, 16]. As the WHO influenza vaccine position paper does not define the “elderly” age group [1], we assumed all persons ≥65 years of age. Finally, we estimated the number of HCWs using the skilled healthcare professional density for the African Region (12.8 per 10 000 population) and for the South-East Asian Region (25.7 per 10 000 population) from WHO Global Health Workforce Statistics [17]. During the first year of life, a child received 2 influenza vaccine doses, while subsequently persons received a single-dose annually [1]. We assumed year-round immunization programs for all vaccines except for influenza vaccines, which were delivered in preseasonal, mass vaccination campaigns of 3 months duration. For this article, we refer to standard, noninfluenza immunization programs as “routine” immunization and influenza immunization programs as pre-seasonal, mass-vaccination “campaigns.”

**Table 1.** Routine and Influenza Vaccines Schedules, and Cold Storage Volume per Dose

	Schedule	Cold Storage Tertiary Packaging volume per dose (mL)	Cold Storage Secondary Packaging Volume per dose (mL)
Routine infant immunization			
Bacille Calmette-Guerin	Birth dose	4.98	1.44
Hepatitis B	Birth dose	12.56	2.86
Diphtheria-tetanus-pertussis-hepatitis B-Haemophilus influenzae type b (pentavalent)	6, 10, and 14 weeks	16.70	3.06
Polio (oral)	6, 10, and 14 weeks	6.22	1.40
Polio (inactivated)	6 weeks	23.95	4.00
Pneumococcal (conjugate)	6, 10, and 14 weeks	36.28	3.60
Rotavirus	6 and 10 weeks	49.45	46.30
Measles-rubella	9–12 months, 13–24 months	9.84	2.11
Tetanus-diphtheria	13–24 months	9.47	2.38
Meningococcal A (conjugate) (African country only)	13–24 months	9.84	2.11
Yellow fever (African country only)	9–12 months	3.59	2.99
Japanese encephalitis (South-East Asian country only)	9–12 months	39.4	4.2
Routine children immunization			
HPV^4^ (girls only)	2 doses from 9 to 14 years	7.61	4.84
Tetanus-diphtheria	9–14 years	9.47	2.38
Influenza immunization			
Risk groups: children <5 years, pregnant women, ≥65 years, healthcare workers, chronic diseases	2 doses, 1 month apart for first year of life, and 1 dose annually thereafter	7.22 (multidose vial)	5.40 (multidose vial)
		87.3 (single dose vial)	18.4 (single dose vial)
		111.8 (prefilled syringe)	86.5 (prefilled syringe)

Vaccines and schedules are from World Health Organization (WHO) policy statements and immunization tables [1]. Tertiary and secondary packaging volumes per dose from WHO Prequalified Vaccines Database. Human papillomavirus (HPV) vaccination programs generally target girls only aged 9–14 years [18]. We assumed the full HPV immunization series was given to girls 9 years of age.

### Immunization System Assumptions

We identified WHO prequalified routine and seasonal influenza vaccines and recorded their secondary and tertiary packaging volumes (Table 1) [18]. Vaccines (and diluents, for reconstituted products) are produced by the manufacturer in vials or other primary packaging that are then packed together in labeled boxes called “secondary packaging” [18]. Products in secondary packaging are packed in insulated boxes used for shipping called “tertiary packaging” [18]. Vaccines are maintained in tertiary packaging at national levels, which can be 10 times greater than secondary packaging volumes. At subnational levels, excess packaging is removed and vaccines are stored in secondary packaging volumes [19]. We used multidose vials, when available, preferentially choosing 10-dose vials.

We made immunization system assumptions based on WHO guidance (Supplementary Table 2) [11, 19–24]. We assumed 90% routine vaccination coverage and 75% influenza vaccination risk group coverage [25]. We used 25% vaccine wastage (doses that are lost or unused) rates [22]. For routine immunization, our assumptions for reserve stock (excess supply in case of increased demand or stock-outs) differed by immunization system level: 3 months at the national level, 1 month at the district and regional levels, and one-half month at the health facility levels [11, 22]. For influenza immunization reserve stock, we used 10% [26]. We assumed that supply intervals for routine immunization were 3 months and that all influenza vaccines were delivered in a single shipment [21].

We next accessed estimates of vaccine cold storage capacity from WHO African Region countries [9]. These data were previously published as volume per population of children aged two years and younger, with values for 25th percentile, 50th percentile, 75th percentile, and upper range for countries in the region that were eligible for support from Gavi, the Vaccine Alliance [26]. We applied these standardized storage capacity values to our simulated African country population to estimate the available vaccine cold storage capacity. No similar data were available for the South-East Asian Region.

### Analysis

**Figure 1.**
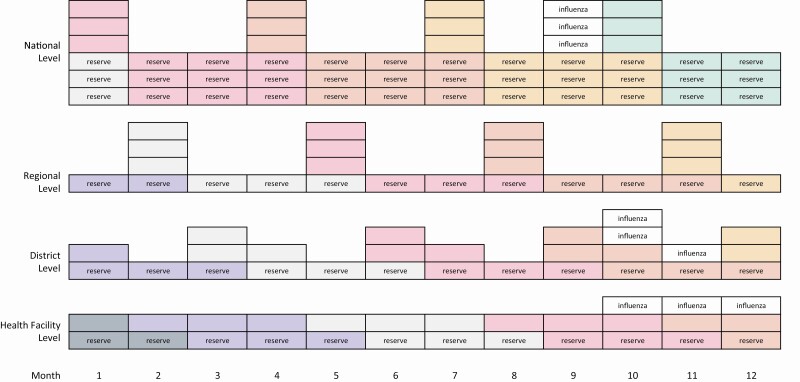
Vaccine flow-down schema. Schema describes vaccine storage and movement through a country from national level to health facility level. Each box represents storage for routine immunization or for an influenza vaccination campaign. Each color represents the flow-down of a tranche of vaccines after each resupply. Reserve stock and influenza vaccines are labeled. Each box represents 1 month supply, except for the reserve stock at the Facility level, which is one-half month supply.

We assumed that vaccines would be distributed according to a vaccine flow-down schema (Figure 1) [9]. The schema assumes 4 levels within the immunization system (national, regional, district, and health facility) and allocates routine and influenza vaccines within each level according to our study assumptions. We calculated and compared the monthly doses and cold storage volumes for routine and influenza immunization programs. Analyses of vaccine volumes used the month from the schema with the highest storage volume for national and subnational levels. For the monthly comparisons and for time series analyses, we assumed constant routine vaccine demand over the year and constant influenza vaccine demand over the 3-month campaign. To assess immunization program workloads, we calculated vaccine doses delivered per vaccinator. We estimated the number of vaccinators by using median nurse density per capita for countries in the WHO African Region (6.9 per 10 000 population) or for countries in the South-East Asian Region (16.4 per 10 000 population) multiplied by the simulated country populations and then adjusted by estimates of nurses providing immunization services (43%) [27] and baseline nurse absenteeism (3%) [28]. We compared monthly doses per vaccinator for influenza vaccination target groups to routine immunization baselines. To assess the feasibility of storing influenza vaccines in the African Region country, we compared the anticipated vaccine volumes for routine and influenza immunization to the vaccine cold storage capacity estimates.

We conducted a secondary analysis to assess the impact of single-dose or prefilled syringe vaccine formulations on vaccine storage in the simulation countries. We used secondary and tertiary volumes for WHO prequalified single-dose vial influenza vaccines [18]. As there is no WHO prequalified prefilled syringe influenza vaccine, we used product characteristics for FluBlok (personal communication, Global Medical Information, Sanofi Pasteur Inc). For these single-use products, we decreased wastage assumptions to 5% (Supplementary Table 2) [22].

All analyses used standard spreadsheet software, including the made-for-purpose “WHO Vaccine Volume Calculator 2012” (Microsoft Corp, Redmond, WA, US) [24].

## RESULTS

### Immunization Target Groups

We simulated an African and an Asian country of 20 million population each (Table 2). The number of people in influenza risk groups was greater in the African country than in the Asian country for children <5 years (3 139 586 vs 1 791 191) and pregnant women (766 588 vs 402 518), although the African country had fewer people than the Asian country for persons ≥65 years (611 807 vs 1 275 885), persons with chronic diseases (2 079 729 vs 2 976 640), and HCWs (25 580 vs 49 200).

**Table 2.** Age Distribution and Influenza Risk Group Size in Simulated Countries

	African Country		South-East Asian Country	
Age group, y	Population	% of total	Population	% of total
<5	3 139 586	15.7%	1 791 191	9.0%
5–9	2 828 076	14.1%	1 884 629	9.4%
10–14	2 500 943	12.5%	1 901 534	9.5%
15–19	2 128 524	10.6%	1 906 139	9.5%
20–24	1 817 105	9.1%	1 781 715	8.9%
25–29	1 559 398	7.8%	1 672 212	8.4%
30–34	1 331 654	6.7%	1 581 780	7.9%
35–39	1 109 669	5.5%	1 431 046	7.2%
40–44	898 313	4.5%	1 278 156	6.4%
45–49	717 631	3.6%	1 124 939	5.6%
50–54	571 040	2.9%	940 571	4.7%
55–59	448 210	2.2%	782 177	3.9%
60–64	338 046	1.7%	648 025	3.2%
65–69	238 867	1.2%	500 675	2.5%
70+	372 940	1.9%	775 209	3.9%
Total	20 000 000		20 000 000	
Influenza risk groups	Population	% of total	Population	% of total
<5 y	3 139 586	15.7%	1 791 191	9.0%
Pregnant women	766 588	3.8%	402 518	2.0%
≥65 y	611 807	3.1%	1 275 885	6.4%
Chronic diseases	2 079 729	10.4%	2 976 640	14.9%
HCWs	25 580	0.1%	49 200	0.2%

Age distributions from each World Health Organization (WHO) Region were applied to the simulated population of 20 000 000. Further details about the calculations of influenza risk groups are in Supplementary Tables 1 and 2.

Abbreviation: HCW, healthcare workers.

### Routine and Influenza Vaccine Doses

**Figure 2.**
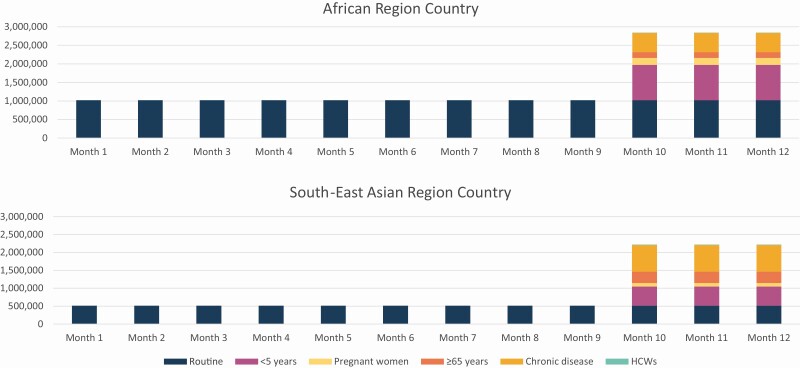
Monthly vaccine doses for routine and influenza vaccination programs in simulated African and South-East Asian countries. We assumed equal distribution of routine vaccines (over twelve months) and influenza vaccines (over a 3-month preseasonal campaign).

The monthly routine doses delivered in the African country was 1 020 931 and in the Asian country was 513 109. In the African country, during influenza vaccination campaigns, the monthly doses delivered would increase depending on the target group receiving the influenza vaccines: by 950 289 (93.1.%) for <5 years, 191 647 (18.8.%) for pregnant women, 152 952 (15.0%) for ≥65 years, 519 932 (50.9%) for chronic diseases, and 6395 (0.6%) for HCW (Table 3 and Figure 2). In the Asian country, influenza vaccination would increase monthly doses delivered during vaccination campaigns by 532 163 (103.7%) for <5 years, 100 630 (19.6%) for pregnant women, 318 971 (62.2.%) for ≥65 years, 744 160 (145.0%) for chronic diseases, and 12 300 (2.4%) for HCW (Table 3 and Figure 2).

**Table 3.** Monthly Vaccine Doses, Doses per Vaccinator, National-level Volumes, and Subnational-level Volumes for Routine and Influenza Vaccination Programs

African Country	Routine Vaccines	<5 years Influenza Vaccines	Pregnant Women Influenza Vaccines	≥65 years Influenza Vaccines	Chronic Diseases Influenza Vaccines	Healthcare Workers Influenza Vaccines
Doses in 1 month during influenza vaccination campaign	1 020 931	950 289	191 647	152 952	519 932	6395
% of routine	…	93.1%	18.8%	15.0%	50.9%	0.6%
Doses in 1 month during influenza vaccination campaign per vaccinator	177.4	165.1	33.3	26.6	90.3	1.1
% of routine	…	93.1%	18.8%	15.0%	50.9%	0.6%
National-level maximum monthly vaccine volume, L	139 161	28 302	5708	4555	15 485	190
% of routine	…	20.3%	4.1%	3.3%	11.1%	0.1%
Subnational-level maximum monthly vaccine volume, L	57 472	21 168	4269	3407	11 581	142
% of routine	…	36.8%	7.4%	5.9%	20.2%	0.2%
South-East Asian Country	Routine vaccines	<5 years influenza vaccines	Pregnant women influenza vaccines	≥65 years influenza vaccines	Chronic diseases influenza vaccines	Healthcare workers influenza vaccines
Doses in 1 month during influenza vaccination campaign	513 109	532 163	100 630	318 971	744 160	12 300
% of routine	…	103.7%	19.6%	62.2%	145.0%	2.4%
Doses in 1 month during influenza vaccination campaign per vaccinator	89.1	92.5	17.5	55.4	129.3	2.1
% of routine	…	103.7%	19.6%	62.2%	145.0%	2.4%
National-level maximum monthly vaccine volume, L	77 067	15 849	2997	9500	22 163	366
% of routine	…	20.6%	3.9%	12.3%	28.8%	0.5%
Subnational-level maximum monthly vaccine volume, L	29 619	11 854	2242	7105	16 576	274
% of routine	…	40.0%	7.6%	24.0%	56.0%	0.9%

Monthly influenza vaccine doses were calculated assuming a 3-month preseasonal influenza vaccination campaign. Immunization assumptions are in Supplementary Tables 1 and 2.

### Routine and Influenza Vaccination Workloads

**Figure 3.**
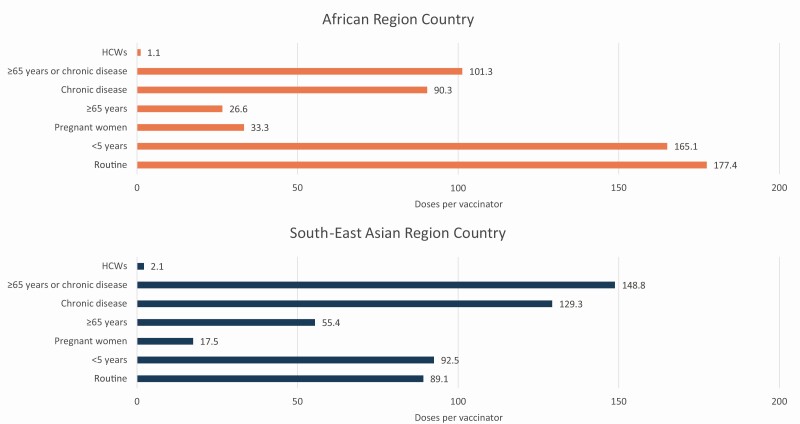
Monthly routine and influenza vaccination program doses per vaccinator in simulated African and South-East Asian countries. Monthly influenza vaccine doses were calculated assuming a 3-month preseasonal influenza vaccination campaign. The number of vaccinators was calculated by multiplying the median nurse density per capita for the African Region (12.8 per 10 000 population) and the South-East Asian Region (25.7 per 10 000 population) by the simulated country populations and then adjusting by estimates of nurses providing immunization services (45%) and baseline nurse absenteeism (3%). Abbreviation: HCW, healthcare workers.

The monthly routine vaccine doses delivered per vaccinator was 177.4 in the African country and 89.1 in the Asian country (Table 3 and Figure 3). Targeting HCW for influenza vaccination would have minimal impact on workload, increasing monthly doses during influenza vaccination campaigns per vaccinator by 1.1 for the African country and by 2.1 for the Asian country. Targeting other influenza risk groups would increase monthly doses per vaccinator during influenza vaccination campaigns in the African country from 26.6 (≥65 years) to 165.1 (<5 years) and in the Asian country from 17.5 (pregnant women) to 129.3 (chronic diseases).

### Routine and Influenza Vaccine Cold Storage Volumes

Vaccine cold storage capacity requirements change through the year depending on supply intervals and distribution. At the national level, the maximum monthly routine vaccine volume was 139 161 L for the African country and 77 067 L for the Asian country (Table 3). Influenza vaccination of HCW had a small impact on maximum monthly vaccine volumes at national and subnational levels for both countries, and this risk group is excluded from volume ranges mentioned below. At the national level, influenza vaccination of other risk groups would increase cold storage capacity needs in the African country from 4555 L (3.3%) for ≥65 years to 28 302 L (20.3%) for <5 years and in the Asian country from 2997 L (3.9%) for pregnant women to 22 163 L (28.8%) for chronic diseases. At the subnational level, influenza vaccination of risk groups would increase cold storage capacity needs in the African country from 3407 L (5.9%) for ≥65 years to 21 168 L (36.8%) for <5 years and in the Asian country from 2242 L (7.6%) for pregnant women to 16 576 L (56.0%) for chronic diseases.

### Impact on Vaccine Storage Capacities in the African Country

**Figure 4.**
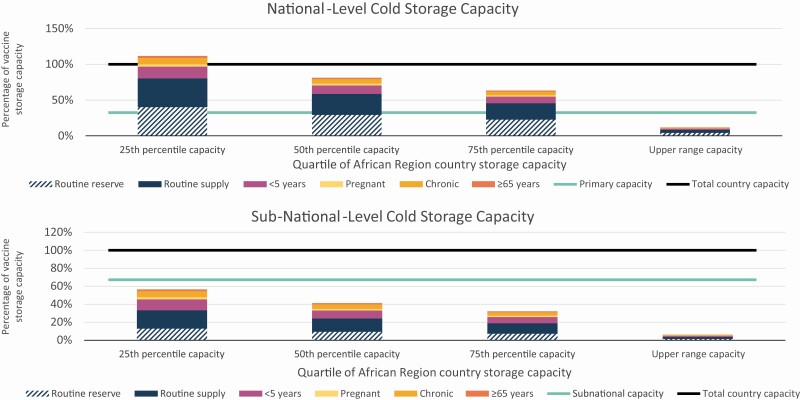
Maximum national-level and subnational-level monthly routine and influenza vaccine volumes as a proportion of total country storage capacity, by quartile of African Region country storage capacity. Vaccines are distributed according to a vaccine flow-down schema (Figure 1).These analyses used the month from the schema with the highest storage volume for national and subnational levels. Cold storage capacity data were accessed as volume per population of children two years and younger, with values for 25th percentile, 50th percentile, 75th percentile, and upper range. These analyses applied the standardized capacities to the country population.

We compared maximum monthly vaccine volumes to quartiles and upper-range cold storage capacities of countries in the African Region (Table 4 and Figure 4). The national-level cold storage capacities were 56 519 L (25th percentile), 77 498 L (50th percentile), 99 424 L (75th percentile), and 524 142 L (upper range). Routine vaccine volumes exceeded national-level storage capacity for all but the upper range country capacity. The percentage of total national-level stores required by influenza vaccines targeting risk groups by quartile of African Region country capacities ranged from 5.4% to 50.1% for <5 years, 1.1% to 10.1% for pregnant women, 3.0% to 27.4% for chronic diseases, 0.9% to 8.1% for ≥ 65 years, and < 0.1% to 0.3% for HCW. The subnational-level cold storage capacities were 116 640 L (25th percentile), 159 935 L (50th percentile), 205 185 L (75th percentile), and 1 081 685 L (upper range). The percentage of total subnational-level stores required by influenza vaccines targeting risk groups by quartile of African Region country capacities ranged from 1.3% to 12.1% for <5 years, 0.3% to 2.4% for pregnant women, 0.7% to 6.6% for chronic diseases, 0.2% to 1.9% for ≥65 years, and <0.1% to 0.1% for HCW.

**Table 4.** Maximum Monthly Storage Volume for Routine and Influenza Vaccines by Quartile of African Region Country Storage Capacity

		25th percentile capacity	50th percentile capacity	75th percentile capacity	Upper Range capacity
Country vaccine storage capacity	% of total country capacity	Vaccine volume, L	Vaccine volume, L	Vaccine volume, L	Vaccine volume, L
Total country capacity	100.0%	173 159	237 433	304 609	1 605 826
National level	32.6%	56 519	77 498	99 424	524 142
Subnational level	67.4%	116 640	159 935	205 185	1 081 685
National-level maximum monthly vaccine volumes	Vaccine volume (L)	% of national-level capacity	% of national-level capacity	% of national-level capacity	% of national-level capacity
Routine vaccines	139 161	246.2%	179.6%	140.0%	26.6%
<5 years influenza vaccines	28 302	50.1%	36.5%	28.5%	5.4%
Pregnant influenza vaccines	5708	10.1%	7.4%	5.7%	1.1%
Chronic diseases influenza vaccines	15 485	27.4%	20.0%	15.6%	3.0%
≥65 influenza vaccines	4555	8.1%	5.9%	4.6%	0.9%
HCW influenza vaccines	190	0.3%	0.2%	0.2%	<0.1%
Subnational-level maximum monthly vaccine volumes	Vaccine volume (L)	% of subnational-level capacity	% of subnational-level capacity	% of subnational-level capacity	% of subnational-level capacity
Routine vaccines	57 472	49.3%	35.9%	28.0%	5.3%
<5 years influenza vaccines	21 168	12.1%	8.8%	6.9%	1.3%
Pregnant influenza vaccines	4269	2.4%	1.8%	1.4%	0.3%
Chronic diseases influenza vaccines	11 581	6.6%	4.8%	3.8%	0.7%
≥65 influenza vaccines	3407	1.9%	1.4%	1.1%	0.2%
HCW influenza vaccines	142	0.1%	0.1%	<0.1%	<0.1%

We developed a vaccine flow-down schematic (Figure 1) to depict the routine vaccine doses maintained at each immunization system level by month and used it to calculate the total monthly vaccine doses and volumes stored throughout the immunization system. This table uses the maximum monthly national-level (month 10) and subnational-level (month 11) vaccine storage volumes from the schematic. The overall vaccine storage capacity for Gavi-eligible African Region countries was standardized by dividing by the <2 years country population in 2017. The range and quintiles of these values were calculated and then applied to the simulated African country. We used the median percentage of national-level stores to total stores (32.6%) and health facility stores to total stores (34.9%) from the same data set for our analyses. Immunization assumptions are in Supplementary Tables 1 and 2.

Abbreviation: HCW, healthcare workers.

### Secondary Analysis

We calculated the cold storage volumes of influenza vaccines targeting risk groups using single-dose vials and prefilled syringes. The percentage of influenza vaccine volume increased the same proportion for each target group (Supplementary Table 3). The use of single-dose vials and prefilled syringe vaccines substantially increased influenza vaccine storage volume requirements as compared to multidose vial vaccines for national-level stores (1015.7% and 1300.7%) and subnational-level stores (285.9% and 1345.6%).

## DISCUSSION

We used WHO tools and guidance to estimate the vaccine delivery, workload, and storage requirements for influenza vaccination programs in simulated African and Asian countries. Our study indicates that conditions are more favorable for programs targeting pregnant women or HCWs than other risk groups. These groups have established platforms through antenatal care and healthcare infrastructures that could be modified or strengthened to include influenza immunization; the anticipated increased number of doses, doses per vaccinator, and cold storage volumes are small for these risk groups compared to others. Our findings indicate that the infrastructure can more easily support recent global efforts to increase influenza vaccine delivery to pregnant women and HCWs to strengthen immunization platforms and pandemic preparedness efforts [29]. Expansion of country immunization programs to include influenza vaccination of children <5 years would use existing routine immunization infrastructures and established immunization contacts, but it would result in around 2-fold increases in doses delivered and substantially higher cold storage requirements during influenza vaccination campaign months. In most of WHO African and South-East Asian Region countries, routine immunization contacts beyond 14 years of age have not been established [4]. Targeting persons with chronic diseases or ≥65 years of age would require new immunization contacts as well as substantial logistics and cold storage capacity above what is needed for existing routine immunization programs. These challenges may be ameliorated by future influenza vaccines if they were to provide protection beyond one year [5, 6].

For at least the last 20 years, WHO has advocated for influenza vaccination programs with the dual purpose of preventing influenza morbidity and providing necessary infrastructures for pandemic response [30]. During the 2009 influenza A (H1N1) pandemic, countries with national influenza vaccination programs were more prepared to receive and use donated pandemic vaccines [31]. Other evidence-based preventive services could be integrated with adult vaccination visits and strengthen care delivery overall [32]. Nevertheless, given the limited LMIC adoption of influenza vaccination policies and the specific challenges of influenza vaccine delivery, the onus is on the public health community to demonstrate the value proposition of new influenza vaccine program implementation in LMICs.

Our study provides additional insights into the challenges of introducing influenza vaccines in LICs and LMICs. Programs targeting children <5 years, older adults, or persons with chronic diseases would substantially impact vaccinator workload. Adult-targeted policies would particularly affect the South-East Asian Region which has a much larger adult population. The low number of vaccinators in the WHO African and South-East Asian Regions and the size of most risk groups suggest influenza vaccination campaigns may disrupt other health services. Furthermore, the additional doses required for influenza vaccination campaigns would greatly increase vaccine transportation and storage requirements within the cold chain. Routine influenza immunization offered throughout the year [33], as an alternative to preseasonal campaigns, would still require large increases in vaccine doses and volumes. Year-round influenza immunization programs are rare and would require alternating procurement of Northern and Southern Hemisphere vaccine formulations and stock rotations timed to the resulting staggered product expiration dates [33]. In our analysis of the African country cold storage capacities, we found that national-level stores would require significant expansion to accommodate the large volumes of recommended routine childhood vaccines and influenza vaccines targeting most risk groups. Strategies such as forward deployment of vaccines from national-level stores to subnational-level stores, the removal of vaccines from tertiary packaging for storage at the national level, and careful coordination of timing for routine vaccine resupply could limit the impact of influenza vaccine deliveries on the national-level stores. Although there appears to be sufficient subnational-level storage capacity in the African country, care will be required in the distribution of vaccines through the immunization system. It is impractical for districts or health facilities to store vaccines intended for use outside of their service areas.

Our secondary analysis shows the limited suitability of influenza vaccines in single-dose vial and prefilled syringe presentations for LIC or LMIC immunization programs [23]. Although prefilled syringes have advantages related to the efficiency of use, their cold storage volumes are prohibitive. Some vaccines used in high-income countries do not have multidose vial presentations (including pneumococcal polysaccharide and recombinant herpes zoster vaccines) [4, 34, 35]. Our study shows that these vaccines may pose logistical challenges in LICs and LMICs when they are introduced.

Our analysis has limitations. We did not include maternal tetanus vaccination in routine immunization assumptions given the 6 doses of tetanus-containing vaccine in our childhood schedule. Most African and South-East Asian Region countries do not include tetanus toxoid vaccination in the second year of life (2% and %) or adolescence (6% and 45%) as we did [36], so our calculated routine vaccine doses and volumes are higher even when excluding maternal tetanus. We were unable to find African or South-East Asian Region prevalence estimates for influenza risk factors. Instead, we used estimates of COVID-19 risk factors, which we believe largely overlap [15, 16]. We used influenza vaccine coverage of 75%. Although aligned with global goals for older adults and persons with chronic diseases [25], this coverage estimate is lower than the global goals of 95% for national coverage for many routine pediatric vaccines [37]. We were not able to compare the influenza vaccination volumes to actual country cold-storage capacities in the Asian country. Not all cold storage space can be effectively utilized; we used total cold storage volumes and not effective cold storage volumes which is estimated to be two-thirds of total volumes [21]. In the past, WHO South-East Asian Region national cold-storage capacities were around 3 times the volume of African Region stores [38], but we do not know if this relationship persists.

### Conclusion

Influenza immunization is under-utilized in LICs and LMICs, despite efforts by the public health community to increase implementation and coverage. The introduction of influenza vaccination programs will require large increases in doses delivered outside of existing immunization contacts and vaccine cold storage volumes in LICs and LMICs. Although there have been recent efforts to strengthen cold storage capacities, our analysis of African countries indicates that much work is still needed to ensure national-level stores are sufficient for routine vaccination programs, and more still for influenza vaccination programs. Attempts to raise influenza immunization coverage goals before addressing limitations in public health resources and making substantial investments in strengthening immunization systems are unlikely to succeed. Although we described challenges to influenza vaccination implementation in LICs and LMICs, the greatest obstacle is the need for annual revaccination. Strengthening of the adult immunization platform for influenza and other vaccines is needed, but new influenza vaccines that are affordable, programmatically suitable, and provide multi-year protection are critical to sustainable influenza vaccination programs in low resource settings.

## Supplementary Data

Supplementary materials are available at *Clinical Infectious Diseases* online. Consisting of data provided by the authors to benefit the reader, the posted materials are not copyedited and are the sole responsibility of the authors, so questions or comments should be addressed to the corresponding author.

ciab393_suppl_Supplementary_Materials

## Notes

***Acknowledgments*.** The authors thank Osman David Mansoor for his advice early in the project and John Gilmore for assistance with figure design.

***Financial support*.** This work had no specific funding support. The work of 2 authors (J. R. and J. S. H.) was supported by the Bill & Melinda Gates Foundation (grant number OPP1177124). The work of 1 author (A. J. D.) was supported by National Institute of Allergy and Infectious Diseases (NIAID) at National Institutes of Health (NIH) (grant number T32AI007524). The views expressed herein are solely those of the authors and do not necessarily reflect the views of the Bill & Melinda Gates Foundation or National Institute of Allergy and Infectious Diseases at National Institutes of Health.

***Potential Conflicts of interest.*** J. R. O. reports grants to his institution from NIH for influenza vaccine research and from NIH, GSK, Pfizer, and PATH for other vaccine research; travel support from Seqirus to attend an influenza vaccine Real World Evidence Scientific Advisory Board meeting; payments to serve as an Independent Safety Monitor and to be a member of a data and safety monitoring board from Pharmaron for noninfluenza vaccine research. He is Co-chair of the American Thoracic Society, Assembly on Pulmonary Infections, and Tuberculosis Working Group on Vaccines and Immunization. W. H. C. reports being on a Safety Review Committee for a Flugen vaccine trial and a data and safety monitoring board member for intramural NIH NIAID vaccine trials. He is a voting member of the Centers for Disease Control and Prevention (CDC) Advisory Committee on Immunization Practices. K.M. N. reports grants to her institution from NIH for influenza vaccine research and grants to her institution from GSK, Pfizer, Bill & Melinda Gates Foundation, and PATH for vaccine (noninfluenza) research. She is on the Board of Directors for the National Foundation of Infectious Diseases, is a member of WHO Strategic Advisory Group of Experts on Immunization (SAGE) (all unpaid), and is co-chair of the NIH COVID Vaccine Prevention Network with salary support from NIH. All other authors report no potential conflicts.
