# Supplementary material for: The Operational Feasibility of Vaccination Programs Targeting Influenza Risk Groups in the World Health Organization (WHO) African and South-East Asian Regions
Source: Clin Infect Dis. 2021 May 4;74(2):227–36. doi: 10.1093/cid/ciab393 (PMC8800189; doi:10.1093/cid/ciab393)
Supplement: ciab393_suppl_Supplementary_Materials [file ciab393_suppl_supplementary_materials.docx]

**TITLE:** The operational feasibility of vaccination programs targeting influenza risk groups in the WHO African and South-East Asian Regions

**ONLINE SUPPLEMENT**

Supplemental Table 1. Influenza vaccination target group assumptions

| **Target Group** | **Assumption** |
| --- | --- |
| <5 years influenza vaccine doses | Two doses delivered to persons <1 year of age and one dose for to persons aged 1 through 4 years. Population data from the Institute for Health Metrics and Evaluation. |
| Pregnant women influenza vaccine doses | 1 dose to all pregnant women. The total birth cohort is 85% of the total number of pregnancies during the year (with the remaining 15% of pregnancies lost to miscarriage or stillbirth). Influenza vaccines were distributed evenly across the childbearing years 15 through 49. Population data from the Institute for Health Metrics and Evaluation. |
| ≥65 years influenza vaccine doses | 1 dose for all persons aged 65 years and older. Population data from the Institute for Health Metrics and Evaluation. |
| Chronic diseases influenza vaccine doses | 1 dose for all persons with chronic disease. We could not find estimates for prevalence of influenza risk factors for the two WHO Regions, so we used estimates for COVID-19 risk factors, which are similar. |
| HCWs influenza vaccine doses | One dose for all health workers using density estimates for each region from WHO Global Health Workforce Statistics [1]. |

Supplemental Table 2. Immunization program assumptions

|  | **Routine immunization assumption** | **Influenza immunization assumption** | **Comments** |
| --- | --- | --- | --- |
| **Vaccines** | WHO prequalified vaccines in multidose vial presentation given per WHO recommended schedule. We used 10-dose vials when they were available. | WHO prequalified vaccine in 10 dose vial presentation given in a two vaccination series, one month apart for persons <1 year of age. | For secondary analyses, we used single dose and prefilled syringe vaccine formulations. For single dose vial storage volumes, we used WHO prequalified single dose vial storage volumes. Since no prefilled influenza vaccines are prequalified, we used data provided by Sanofi Pasteur for FluBlok used in the United States. |
| **Packaging** | Tertiary packaging at the national level  Secondary packaging at all subnational levels | Tertiary packaging at national level  Secondary packaging at all subnational levels | National level vaccine volume analyses use total tertiary packaging volume required per dose (the unit for international transport), defined as the volume of the container holding cartons which contain vaccine vials divided by the total doses contained.  Subnational levels vaccine volume analyses use total secondary packaging volume required per dose, defined as the volume of cartons which contain vaccine vials divided by the total doses contained. |
| **Storage Temperature** | 2° to 8°C | 2° to 8°C |  |
| **Coverage** | 90% | 75% of target group | For secondary analysis, we assessed coverage of ≥75% for persons with chronic diseases and persons older than 65 years to meet 2003 WHA resolution goals. |
| **Target groups** | WHO recommended ages | Children <5 years  Pregnant women  Persons ≥65 years  Healthcare workers  Chronic diseases (all ages) |  |
| **Strategy** | Year round | Three month mass influenza vaccination campaign |  |
| **Wastage multidose vials** | 25% | 25% | For single dose presentations, we used 5%. |
| **Reserve stock** | 3 months at national level  1 month at district and regional levels  0.5 months at health facility level | 10% at all levels | In the absence of any buffer stock policy for influenza immunization, we used a 10% buffer in the primary analysis [2]. |
| **Resupply intervals** | 3 months at national, district, and regional levels  1 month at health facility level | One annual supply at national, district, and regional levels  1 month at health facility level | Three month supply interval is common for routine immunization in low resource settings, influenza vaccines were delivered in one pre-seasonal shipment [3]. |
| **Vaccinators** | Median nurse density per capita for countries in each WHO Region multiplied by the simulated country population, and adjusted by the proportion of nurses providing immunization services and estimates of absenteeism at baseline | Same as for routine | Typically, persons delivering vaccines in the region are nurses, but not all nurses engage in the provision of immunization services. The estimates for nurses per capita are from WHO Global Health Workforce Statistics [1]. We estimated the percentage of nurses providing immunization services using survey data from the Organisation for Economic Co-operation and Development [4]. Absenteeism estimates are from observational data from the United States [5, 6]. |

**Supplemental Table 3. Maximum necessary cold chain storage volume per month given 4x yearly supply intervals, routine immunization and influenza vaccination programs by target group and alternative vaccine presentation (single-dose vial or prefilled syringe)**

|  |  | **multi-dose vial** | | | **single-dose vial** | | | **prefilled syringe** | | |
| --- | --- | --- | --- | --- | --- | --- | --- | --- | --- | --- |
|  | **National Level** | **max monthly volume** | **% of routine** | **max monthly volume** | | **% of routine** | **% of multidose volume** | **max monthly volume** | **% of routine** | **% of multidose volume** |
| Sub-Saharan African Country | <5 years | 28,302 | 20.3% | 287,457 | | 206.6% | 1015.7%. | 368,129 | 264.5% | 1300.7%. |
|  | Pregnant women | 5,708 | 4.1% | 57,972 | | 41.7% | 1015.7%. | 74,242 | 53.3% | 1300.7%. |
|  | ≥65 years | 15,485 | 11.1% | 157,277 | | 113.0% | 1015.7%. | 201,415 | 144.7% | 1300.7%. |
|  | Chronic diseases | 4,555 | 3.3% | 46,267 | | 33.2% | 1015.7%. | 59,252 | 42.6% | 1300.7%. |
|  | HCWs | 190 | 0.1% | 1,934 | | 1.4% | 1015.7%. | 2,477 | 1.8% | 1300.7%. |
| South East Asian Country | <5 years | 15,849 | 20.6% | 160,976 | | 208.9% | 1015.7%. | 206,153 | 267.5% | 1300.7%. |
|  | Pregnant women | 2,997 | 3.9% | 30,440 | | 39.5% | 1015.7%. | 38,983 | 50.6% | 1300.7%. |
|  | ≥65 years | 22,163 | 28.8% | 225,104 | | 292.1% | 1015.7%. | 288,278 | 374.1% | 1300.7%. |
|  | Chronic diseases | 9,500 | 12.3% | 96,487 | | 125.2% | 1015.7%. | 123,565 | 160.3% | 1300.7%. |
|  | HCWs | 366 | 0.5% | 3,721 | | 4.8% | 1015.7%. | 4,765 | 6.2% | 1300.7%. |
|  | **Subnational Levels** | **max monthly volume** | **% of routine** | **max monthly volume** | | **% of routine** | **% of multidose volume** | **max monthly volume** | **% of routine** | **% of multidose volume** |
| Sub-Saharan African Country | <5 years | 21,168 | 36.8% | 60,521 | | 105.3% | 285.9% | 284,823 | 495.6% | 1345.6% |
|  | Pregnant women | 4,269 | 7.4% | 12,205 | | 21.2% | 285.9% | 57,441 | 99.9% | 1345.6% |
|  | ≥65 years | 11,581 | 20.2% | 33,113 | | 57.6% | 285.9% | 155,835 | 271.2% | 1345.6% |
|  | Chronic diseases | 3,407 | 5.9% | 9,741 | | 16.9% | 285.9% | 45,843 | 79.8% | 1345.6% |
|  | HCWs | 142 | 0.2% | 407 | | 0.7% | 285.9% | 1,917 | 3.3% | 1345.6% |
| South East Asian Country | <5 years | 11,854 | 40.0% | 33,892 | | 114.4% | 285.9% | 159,501 | 538.5% | 1345.6% |
|  | Pregnant women | 2,242 | 7.6% | 6,409 | | 21.6% | 285.9% | 30,161 | 101.8% | 1345.6% |
|  | ≥65 years | 16,576 | 56.0% | 47,393 | | 160.0% | 285.9% | 223,042 | 753.0% | 1345.6% |
|  | Chronic diseases | 7,105 | 24.0% | 20,314 | | 68.6% | 285.9% | 95,603 | 322.8% | 1345.6% |
|  | HCWs | 274 | 0.9% | 783 | | 2.6% | 285.9% | 3,687 | 12.4% | 1345.6% |

**REFERENCES**

1. World Health Organization. WHO Global Health Workforce Statistics: December 2018 update. Available at: <https://www.who.int/hrh/statistics/hwfstats/en/>. Accessed 28 September 2020.

2. Debellut F, et al. Forecasting demand for maternal influenza immunization in low- and lower-middle-income countries. PLoS One **2018**; 13(6): e0199470.

3. World Health Organization. Guideline for establishing or improving primary and intermediate vaccine stores. Available at: <https://apps.who.int/iris/bitstream/handle/10665/67807/WHO_V-B_02.34_eng.pdf>.

4. International Council of Nurses. The Role of Nurses in Immunisation: a Snapshot from OECD Countries. Available at: <https://www.icn.ch/sites/default/files/inline-files/IMMUNISATION_Report%20%28002%29.pdf>. Accessed June 3, 2020.

5. Chin ET, et al. Healthcare worker absenteeism, child care costs, and COVID-19 school closures: a simulation analysis. medRxiv **2020**.

6. Ticharwa M, et al. Nurse absenteeism: An analysis of trends and perceptions of nurse unit managers. J Nurs Manag **2019**; 27(1): 109-16.
